# From Diagnosis to Treatment of Arrhythmic Mitral Valve Prolapse and Mitral Annular Disjunction

**DOI:** 10.3390/jcdd13100492

**Published:** 2026-10-01

**Authors:** Michalina Dworak, Krzysztof Jaworski, Kacper Karaban, Anna Wałachowska, Ewa Świerżyńska-Wodarska, Marek Misiak, Anna Jargieło, Ilona Michałowska, Rafał Dąbrowski, Łukasz Szumowski, Michał Orczykowski, Magdalena Marczak, Maciej Sterliński

**Affiliations:** 1 Students’ Scientific Society, National Institute of Cardiology, 04-628 Warsaw, Poland; 2 Department of Coronary Artery Disease and Cardiac Rehabilitation, National Institute of Cardiology, 04-628 Warsaw, Poland; 3 1st Department of Arrhythmia, National Institute of Cardiology, 04-628 Warsaw, Poland; 4 Department of Radiology, National Institute of Cardiology, 04-628 Warsaw, Poland; 5 Magnetic Resonance Unit, National Institute of Cardiology, 04-628 Warsaw, Poland

**Keywords:** mitral valve prolapse, mitral annular disjunction, ventricular arrhythmias, ventricular tachycardia, arrhythmic mitral valve prolapse, risk stratification, sudden cardiac death

## Abstract

Mitral valve prolapse (MVP) and mitral annular disjunction (MAD) are common comorbidities that can lead to serious cardiac arrhythmias and sudden cardiac death (SCD). Arrhythmic MVP is characterized by the presence of MVP with frequent (over 5% ventricular extra beats) and/or complex ventricular arrhythmias, such as ventricular tachycardia (VT), non-sustained VT, or ventricular fibrillation in the absence of other causes of arrhythmia, e.g., post-infarction scar, recent ischemia, channelopathy, or cardiomyopathy. The mechanisms of the development of serious cardiac arrhythmias in patients with MVP and MAD are not fully understood. Arrhythmic MVP may be an indication for cardioverter defibrillator implantation. Considering the increased risk of SCD in this group of patients, the pathophysiology of this valvular dysfunction and characteristic deviations in diagnostic tests that indicate a higher risk of SCD are presented. The issue of tailored risk stratification in individual patients is crucial.

## 1. Introduction

Mitral valve prolapse (MVP) and mitral annular disjunction (MAD) are coexisting conditions that may lead not only to hemodynamic disturbances affecting the left atrium and left ventricle but also to potentially life-threatening ventricular arrhythmias and sudden cardiac death (SCD). In autopsy studies of young individuals who experienced SCD, the prevalence of MVP has been reported to range from 4% to 7%. Given the arrhythmogenic potential of MVP and MAD, accurate diagnosis and comprehensive risk stratification are essential, particularly for identifying individuals at increased risk of malignant ventricular arrhythmias and SCD [1,2].

## 2. Literature Search and Selection

This narrative review was informed by a focused appraisal of the literature on MVP, arrhythmogenic mitral valve prolapse (AMVP), MAD, ventricular arrhythmias (VA), and SCD. Literature selection was guided by its relevance to the scope of the review, with particular emphasis on studies investigating the underlying pathophysiological mechanisms, clinical and imaging phenotypes, arrhythmic risk stratification, and contemporary therapeutic strategies. Priority was given to original studies, systematic reviews, and meta-analyses, together with expert consensus documents and position statements issued by relevant professional societies. Seminal earlier studies were retained where they provided important historical context or established concepts that continue to inform contemporary clinical practice.

## 3. Definition of Mitral Valve Prolapse

MVP is defined as systolic displacement of one or both mitral valve leaflets by ≥2 mm into the left atrium beyond the plane of the mitral annulus [3].

## 4. Epidemiology of Mitral Valve Prolapse

MVP was first recognized as a distinct clinical entity in the 1960s and is currently estimated to affect approximately 2–3% of the general population [3]. Although some studies have reported a higher prevalence of MVP among women [4], sex-related differences in prevalence vary across study populations and should be interpreted with caution.

## 5. Diagnosis of Mitral Valve Prolapse

The diagnosis is most commonly established by echocardiography, using parasternal or apical long-axis views; however, complementary imaging modalities, including computed tomography (CT) and cardiac magnetic resonance (CMR), may also be used (Figure 1 and Figure 2) [5].

In most patients, MVP follows a benign course with a favorable prognosis; however, a distinct arrhythmic phenotype associated with VA and SCD has been recognized [3]. Most patients with MVP remain asymptomatic, although some may present with non-specific symptoms, including atypical chest pain, exertional dyspnea, palpitations, and anxiety. On cardiac auscultation, a characteristic mid-systolic click may be present, whereas electrocardiographic assessment may reveal repolarization abnormalities. These clinical manifestations have historically been described as part of the “MVP syndrome” [6,7].

MVP is caused by excessive laxity of the mitral valve leaflets, which leads to leaflet billowing and prolapse (fibroelastic deficiency). Another mechanism involves myxomatous degeneration—changes in leaflet structure such as elongation, thickening, and excessive flexibility, as well as chordal elongation or rupture (Barlow’s disease). MVP may also result from structural damage to the valve apparatus, such as papillary muscle rupture following myocardial infarction or trauma [8].

MVP is the most common cause of clinically significant degenerative mitral regurgitation (DMR). Nevertheless, progression to severe mitral regurgitation occurs relatively infrequently. Untreated severe DMR may lead to progressive LV systolic dysfunction, heart failure, and death. In symptomatic patients with LV dilatation or dysfunction, mitral valve intervention, usually cardiac surgery, is indicated [9].

Figure 3 presents an echocardiogram with typical features of mitral regurgitation caused by MVP.

Due to the heterogeneity of disease etiology, several classification systems have been proposed to categorize the underlying mechanisms and types of MVP. Figure 4 summarizes the principal types of MVP.

Primary MVP may occur sporadically or show familial aggregation. Autosomal dominant inheritance with variable penetrance appears to be more common than X-linked forms [11]. Pathogenic variants in several genes, including FLNA, DCHS1, DZIP1, and PLD1, have been implicated in familial forms of MVP and exhibit heterogeneous patterns of inheritance. It is a relatively common hereditary condition affecting approximately 170 million individuals worldwide.

Secondary MVP is associated with connective tissue disorders, e.g., Marfan syndrome, Ehlers–Danlos syndrome, Loeys–Dietz syndrome, pseudoxanthoma elasticum, osteogenesis imperfecta, and aneurysm–osteoarthritis syndrome [11,12].

Figure 5 presents the recommendations of the European Society of Cardiology (ESC) regarding the management of patients with MVP and the prioritization of diagnostic investigations [13].

## 6. Definition of Mitral Annular Disjunction

In the 1990s, Hutchins analyzed histological cross-sections of the mitral annulus and observed that most mitral valve leaflet attachments were located at the boundary between the left ventricular myocardium and the left atrium. However, in some patients, the attachments were located slightly lower within the ventricle or partially separated from the atrial aspect of the left ventricular myocardium by a tendinous attachment. This anatomical variant was described as “disjunction” [15].

In MAD, the mitral valve insertion zone (annulus) is separated from the adherent ventricular muscle. Studies have found an association between MAD and the coexistence of MVP [15].

## 7. Epidemiology of Mitral Annular Disjunction

Some studies have reported a higher prevalence of MAD among women; however, given the small sample sizes and limited available evidence, this apparent sex-related difference remains uncertain [16,17]. The predisposition of women to the co-occurrence of MVP and MAD may be related to the smaller anatomical size of the mitral annulus [16]. Additionally, MAD is often observed in association with other structural heart diseases, such as cardiomyopathies [18].

## 8. Diagnosis of Mitral Annular Disjunction

Imaging methods are used to diagnose MAD, ranging from echocardiography to more advanced techniques such as CMR. Figure 6 presents examples of MAD with a separation of 4 mm. Accurate assessment of this condition is crucial for evaluating the risk of complications and planning optimal treatment strategies.

Tani et al. classified MAD according to the length of separation. The authors recognized four variants: type 0, in which no MAD is apparent; type I, in which a hypermobile basal left ventricular segment is visible without annular disjunction; type II, in which MAD measures less than 5 mm; and type III, in which MAD exceeds 5 mm.

MAD is most clearly visible in systole, when the posterolateral portion of the LV contracts and the mitral annulus appears to detach from the left ventricular myocardium [19].

MAD diagnosis requires high imaging accuracy. The posterior mitral valve leaflet may be positioned within the atrioventricular junction during diastole, without true anatomical separation between the posterior mitral annulus and the left ventricular myocardium. This phenomenon is referred to as pseudo-MAD.

Lodin et al. conducted a study of 599 patients who underwent mitral valve surgery: repair (485 patients) or replacement (114 patients). MAD was diagnosed in 96 patients, and pseudo-MAD was identified in 39 cases [20].

## 9. Pathophysiology of Arrhythmias in MVP and MAD

The development of cardiac arrhythmias in patients with MVP and MAD is thought to result from a complex interplay of structural, electrical, and autonomic factors. The underlying pathophysiological mechanisms remain incompletely understood and may vary among patients.

Left ventricular fibrosis, which may develop partly as a consequence of repetitive myocardial stretch and mechanical stress associated with abnormal mitral valve motion in MVP and MAD, may contribute to electrical heterogeneity and conduction abnormalities within the ventricular myocardium. Fibrotic tissue may promote regions of slow and heterogeneous conduction, thereby providing a potential substrate for ventricular arrhythmias, including VT and ventricular fibrillation (VF) [21].

In MVP and MAD, structural and functional changes in the heart can affect Purkinje fibers, making them susceptible to premature or irregular transmission. Purkinje fibers play a key role in rapid ventricular impulse propagation, and their dysfunction may facilitate arrhythmogenesis. Abnormal impulse formation and conduction disturbances further contribute to the development of arrhythmias.

Figure 7 presents sinus rhythm with polymorphic premature ventricular complexes occurring in couplets.

Multifocal premature ventricular contractions (PVCs) originating from various parts of the ventricles, especially from areas with fibrosis or other structural abnormalities, may lead to complex ventricular ectopy. In MVP and MAD, the mitral annulus and adjacent structures may serve as sources of ectopic activity. Additionally, patients often exhibit an autonomic imbalance, including altered parasympathetic tone [22,23].

## 10. Arrhythmogenic Aspects of MVP and MAD

If MVP coexists with non-sustained or sustained VT or frequent PVCs, strategies for SCD prevention and symptom-oriented management should be considered. The presence of phenotypic features of arrhythmic MVP, such as redundant mitral valve leaflet tissue, MAD, and T-wave inversion, should prompt extended electrocardiographic monitoring to allow a detailed assessment of ventricular arrhythmias [24].

Studies have shown that MVP predisposes patients to atrial and complex ventricular arrhythmias. In the adult population, the annual risk of sudden death or unexplained syncope ranges from 0.2% to 1.9% [25].

Arrhythmic MVP is defined as the presence of MVP and frequent ventricular ectopy (>5% of ventricular beats) and/or complex ventricular arrhythmias, including VT and VF, in the absence of other causes of arrhythmia, such as post-infarction scarring, ischemic heart disease, channelopathies, or cardiomyopathies. It is recommended that all patients diagnosed with MVP undergo 24 h Holter ECG monitoring and transthoracic echocardiography as a part of a comprehensive evaluation. Some patients may require prolonged ECG monitoring to improve the detection of ventricular arrhythmias. Patients with MVP should also undergo risk stratification for SCD [26]. A female predominance has been reported in some cohorts of patients with arrhythmic MVP and life-threatening ventricular arrhythmias, with women accounting for approximately 70–90% of cases in selected studies [20]. However, evidence supporting sex-specific differences in arrhythmic risk remains limited, and female sex has not been conclusively established as an independent predictor of malignant ventricular arrhythmias or SCD.

All patients with arrhythmic MVP who have experienced sustained ventricular arrhythmias should undergo CMR imaging at least once. CMR is also appropriate and diagnostically useful in patients with MVP who have at least one high-risk factor, such as non-sustained ventricular tachycardia (nsVT) or unexplained syncope. CMR can detect late gadolinium enhancement (LGE), which indicates myocardial fibrosis (Figure 8), a potential substrate for arrhythmias [14].

Chronic mechanical stress on the myocardium promotes fibrosis, which is particularly evident in the basal inferolateral segments of the left ventricle. Fibrotic structural changes contribute to the development of arrhythmias in patients with AMVP. Echocardiography is the primary imaging modality for assessing the severity of MVP and valvular morphology; however, CMR plays a central role in arrhythmic risk stratification, particularly by enabling accurate visualization of myocardial fibrosis [27].

The prognostic value of CMR extends beyond the mere presence or absence of LGE. The extent and distribution of replacement fibrosis may provide additional insight into the arrhythmogenic substrate, particularly when the papillary muscles or the basal inferolateral or inferior segments of the LV are involved. Quantitative assessment of LGE burden may therefore improve phenotypic characterization and potentially refine arrhythmic risk stratification; however, standardized quantification methods and validated prognostic thresholds for fibrosis burden in MVP are still lacking [27].

Papillary muscle fibrosis appears to be particularly relevant in AMVP. Repetitive mechanical stress associated with the prolapsing mitral valve apparatus may lead to papillary muscle injury and subsequent replacement fibrosis, whereas the papillary muscles themselves are frequent sites of origin for ventricular ectopy and tachyarrhythmias [28].

Essayagh et al. demonstrated an association between papillary muscle fibrosis, MAD, and nsVT in patients evaluated using 3-T CMR [29]. Lim et al. specifically investigated papillary muscle LGE and its association with ventricular arrhythmias [30].

Accordingly, the presence of LGE within the papillary muscles may indicate an underlying arrhythmogenic substrate. As conventional bright-blood LGE sequences may have limited sensitivity for detecting fibrosis in this region, dark-blood LGE techniques may improve the visualization and detection of papillary muscle fibrosis [31].

Beyond conventional LGE imaging, parametric mapping provides additional insight into diffuse myocardial remodeling. Native T1 mapping and extracellular volume fraction (ECV) enable the detection of diffuse interstitial fibrosis that may remain undetected by conventional LGE. Increased native T1 and ECV values have been reported in patients with MVP even in the absence of overt LGE, while higher ECV has been associated with a greater burden of ventricular arrhythmias, suggesting a potential role for quantitative tissue characterization in arrhythmic risk assessment [27].

A multiparametric CMR approach integrating cardiac morphology; MAD assessment; the presence, distribution, and extent of LGE; papillary muscle involvement; and quantitative tissue characterization may provide a more comprehensive assessment of the arrhythmogenic substrate. Nevertheless, parametric mapping should currently be regarded as a complementary rather than an established stand-alone risk marker, as standardized acquisition protocols and validated prognostic thresholds remain lacking.

Thus, while LGE represents the most established CMR marker of myocardial fibrosis in AMVP, the incremental prognostic value of fibrosis burden, papillary muscle involvement, and parametric mapping remains to be prospectively validated.

Given the multifactorial nature of arrhythmic risk in MVP, comprehensive risk stratification should integrate clinical, electrocardiographic, echocardiographic, CMR, and genetic findings. The principal markers associated with increased arrhythmic risk are summarized in Table 1.

Data on the prevalence and types of sustained ventricular arrhythmias in AMVP remain limited. Data on VF as the initiating arrhythmia are derived from resuscitation records. Fast monomorphic VT may degenerate into VF; therefore, data collected during resuscitation may lead to the overdiagnosis of VF.

Sabbag et al., in their latest report, presented 278 arrhythmic episodes in 225 patients. The most common arrhythmia was VF, 65%, followed by still frequent, fixed monomorphic ventricular tachycardia (SMVT), 26%. In a subset of 140 episodes with available ECG recordings, short-coupled premature ventricular complexes were identified as triggers in 25% of cases. In nearly half of the cases, arrhythmias were pause-dependent (49%), more frequently leading to VF than to SMVT [32].

Genetic studies suggest that in younger patients, arrhythmias may originate from the papillary muscles, whereas in older individuals, the myocardium is more likely to support macro-reentrant ventricular circuits [33].

Patients who developed VF or polymorphic VT were younger than those with SMVT and more frequently exhibited LGE within the papillary muscles, whereas in patients with SMVT, fibrosis was more commonly located within the ventricular myocardium. In addition, a family history of SCD was more frequently observed in the SMVT group [32].

In most patients, the prognosis of MVP is primarily related to the severity of mitral regurgitation and its consequences for the left ventricle, including progressive dilatation and systolic dysfunction. Although severe ventricular arrhythmias are relatively uncommon, they are associated with a significantly increased risk of SCD, regardless of the severity of MVP [34,35].

The severity of complications, such as atrial fibrillation and ventricular arrhythmias, may depend on the extent of MAD. In addition, MAD may be considered a predictor of adverse clinical outcomes or a poorer prognosis in patients with valvular heart disease and other cardiac conditions [36].

## 11. Sudden Cardiac Death in MVP and MAD Patients

MVP has been identified in approximately 6.6% of patients presenting with unexplained cardiac arrest due to idiopathic ventricular fibrillation.

Alqarawi et al. analyzed 571 cases of SCD, defining unexplained cardiac arrest as cardiac arrest occurring in patients with a normal left ventricular ejection fraction, in the absence of coronary heart disease or evident ECG abnormalities. In this group, echocardiography was used to identify MVP. Patients were divided into two groups: those diagnosed with idiopathic VF and those with other diagnoses.

Among 571 patients in whom the cause of cardiac arrest was initially unclear, 34 (6%) had MVP. The prevalence of MVP was significantly higher in the idiopathic VF group (6.6%) than in patients with other diagnoses (2.4%). Bileaflet MVP (Figure 1) was strongly associated with arrhythmic MVP, occurring in 78% of patients with this diagnosis, compared with 12.5% of patients with other diagnoses. During more than three years of follow-up, 21.1% of patients with arrhythmic MVP received appropriate implantable cardioverter defibrillator (ICD) therapies [37].

In another study, patients with isolated MVP who survived SCA exhibited a higher prevalence of MAD, the Pickelhaube sign, and systolic atrial-directed notch (SADN) > 2 mm, a novel echocardiographic parameter, compared to those with SCA and non-isolated MVP without other defined structural heart diseases. In addition, SCA survivors with isolated MVP had higher absolute values of longitudinal strain in the mid and basal segments of the inferolateral and inferior left ventricular walls [38].

Malignant arrhythmias frequently recur in patients with MVP who have survived SCA. Studies have shown an increased risk of SCD among patients with MVP, accompanied by characteristic abnormalities on diagnostic testing. These abnormalities are summarized in Table 2 [38].

**Table 2:** Characteristic abnormalities in diagnostic tests associated with an increased risk of sudden cardiac death in patients with MVP.

Diagnostic Domain	Risk-Associated Finding	References
**ECG**	Biphasic or inverted T waves in the inferior leads (II, III, aVF)	[28,39]
	QT prolongation	[14]
	Fragmented QRS complexes	[14,40]
**Echocardiography**	Pickelhaube sign—peak systolic lateral mitral annular velocity ≥ 16 cm/s (Figure 9)	[33,34]
	Bileaflet mitral valve prolapse	[41]
**CMR**	Focal fibrosis involving the papillary muscles and the basal inferior LV segment	[2,33]
	Late gadolinium enhancement (LGE)	[2,33]
**Genetics**	FLNA-associated MVP phenotype, including congenital and degenerative abnormalities of the mitral valve apparatus	[31,42]
	FLNA variants potentially associated with an arrhythmogenic phenotype	[31,42]
**Morphological features**	Mitral annular disjunction (MAD) and severe myxomatous degeneration with thick, hyperelastic mitral valve leaflets	[40]

**Figure 9:**
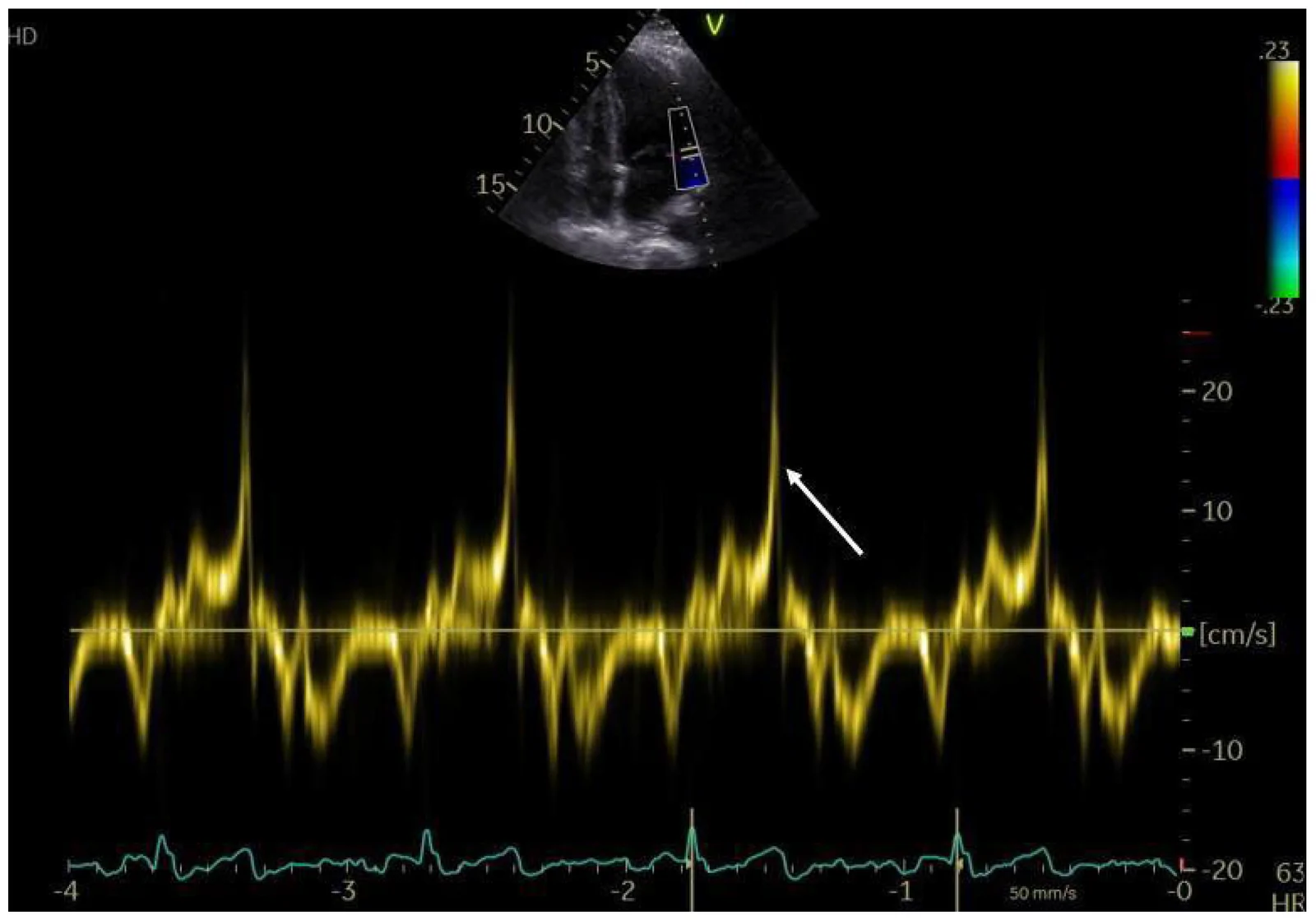
TTE— tissue-doppler imaging, “Pickelhaube sign”—a peak systolic lateral mitral annulus velocity ≥ 16 cm/s (white arrow).

Studies have confirmed that MAD is frequently diagnosed in patients with cardiac arrhythmias [16,39]. One study reported that ventricular arrhythmias (VAs), such as nsVT and VT, or aborted cardiac arrest occurred in 34% of patients with MAD. In addition, 71% of patients in this study reported palpitations, 41% experienced weakness, and 13% experienced syncope [39].

There are several prognostic markers that may indicate a higher risk of VA in patients with MAD. These include younger age, papillary muscle fibrosis, reduced left ventricular ejection fraction (LVEF), and a mitral annular disjunction distance of more than 8.5 mm. Sudden cardiac death may occur in up to 3.8% of patients with MAD. Therefore, a diagnosis of MAD syndrome should be considered in younger patients without other identifiable causes of ventricular arrhythmias and in those presenting with unexplained syncope or presyncope [43].

However, although MAD has been consistently associated with ventricular arrhythmias and adverse arrhythmic phenotypes, its independent prognostic value remains incompletely defined. Proposed MAD thresholds, including a disjunction distance of >8.5 mm, have not been prospectively validated and should therefore not be interpreted as stand-alone criteria for predicting malignant ventricular arrhythmias or SCD.

## 12. Evidence Gaps and Unresolved Controversies

Current evidence regarding arrhythmic MVP is derived predominantly from observational cohorts, retrospective studies, imaging studies, and expert consensus, with relatively limited prospective and randomized data. The association of complex ventricular arrhythmias, unexplained syncope, and myocardial fibrosis with the arrhythmogenic phenotype is relatively well established; however, the independent prognostic value of individual morphological and imaging markers remains less certain. In particular, MAD, bileaflet prolapse, the Pickelhaube sign, papillary muscle fibrosis, LGE burden, and abnormalities detected by parametric mapping should currently be interpreted as components of a multiparametric risk profile rather than as stand-alone predictors of SCD. Similarly, evidence guiding primary-prevention ICD implantation and interventions in patients without conventional indications for mitral valve surgery remains largely based on expert consensus and observational data. Consequently, prospective multicenter studies with standardized phenotyping and predefined arrhythmic endpoints are required to validate risk markers and determine clinically meaningful thresholds for therapeutic decision-making.

## 13. Therapeutic Approach to Arrhythmias Induced by MVP and MAD

### Drug Therapy

Data on the use of antiarrhythmic drugs (AADs) to prevent recurrent VT are limited to small observational studies and registries, and the overall effectiveness of these drugs remains limited [14].

In patients with structural heart disease, beta-blockers are the mainstay of pharmacotherapy for VA. Data from a retrospective registry indicate that chronic beta-blocker therapy in patients with VF or VT is associated with improved long-term prognosis during a 3-year follow-up period.

Amiodarone, especially in combination with a beta-blocker, may reduce the risk of VT recurrence. However, its adverse effects, including thyroid dysfunction and hepatic and pulmonary toxicity, should be considered. A meta-analysis including 3828 patients from 22 randomized trials showed that amiodarone reduced the risk of VT recurrence by 70% compared with standard optimal therapy. The risk of appropriate ICD interventions was also reduced in patients with an implanted device [44].

Amiodarone and class I antiarrhythmic drugs were associated with a lower rate of VT recurrence than sotalol [14,45]. The addition of flecainide to a beta-blocker or sotalol may be beneficial, although this has been demonstrated only in a small cohort of patients [14].

A meta-analysis of RCTs reported that the use of amiodarone may be associated with an increased risk of death. However, it remains unclear whether this association is attributable to the adverse effects of amiodarone or to the greater disease severity of the patients receiving it.

The OPTIC study, which included a one-year follow-up of patients with ICDs for secondary prevention, reported that the combination of amiodarone and a beta-blocker was more effective in reducing the risk of ICD discharge than beta-blocker monotherapy or sotalol therapy. Sotalol monotherapy was more effective than beta-blocker monotherapy. QT prolongation was observed following a single low dose of sotalol [44].

Sotalol was effective in preventing inducible VT but did not reduce the occurrence of clinically significant arrhythmias [14,46].

The effectiveness of other adjunctive antiarrhythmic drugs, such as mexiletine, procainamide, quinidine, and flecainide, depends on the underlying structural heart disease and the type of arrhythmia. These drugs are not universally appropriate for all patients, primarily because of their differing safety profiles [44].

Importantly, much of the evidence supporting antiarrhythmic drug therapy is extrapolated from broader populations with structural heart disease or ventricular arrhythmias rather than being derived specifically from patients with AMVP. Consequently, pharmacological treatment in AMVP remains largely symptom- and arrhythmia-oriented, and there is currently no evidence that antiarrhythmic therapy modifies the underlying disease substrate or prevents SCD in this specific population.

The presence of structural heart disease limits the available therapeutic options. Non-pharmacological therapies, including ICD implantation and ablation, continue to play a key role in the treatment of ventricular arrhythmias [44].

## 14. Interventional Cardiology

### 14.1. Implantable Cardioverter Defibrillator

Indications for ICD implantation for primary and secondary prevention also apply to patients with MVP. Guidelines recommend ICD implantation for primary prevention in patients with symptomatic heart failure and an LVEF ≤ 35% despite 3 months of optimal medical therapy. However, it remains unclear how many patients with MVP/degenerative MR meet this criterion, as an LVEF within this range is uncommon in this population [14].

Primary-prevention ICD implantation remains challenging, as no randomized trials have confirmed its benefit in any subgroup of patients with MVP. The level of evidence supporting ICD implantation differs substantially between secondary and primary prevention. Secondary-prevention ICD implantation after SCA, VF, or sustained VT in the absence of reversible causes is supported by established guideline-based principles. In contrast, evidence for primary-prevention ICD implantation specifically in AMVP remains limited, as no randomized trials have demonstrated a survival benefit in this population. Current recommendations for selected high-risk AMVP phenotypes are therefore largely based on expert consensus and extrapolation from broader populations at risk of SCD.

In patients with AMVP undergoing CMR, particularly in the presence of LGE, careful attention should be paid to the structural evaluation of the heart. MVP-associated ventricular arrhythmias should not be classified as benign or idiopathic.

All patients with MVP should undergo risk stratification for SCD. The authors present a number of imaging, echocardiographic, and electrocardiographic parameters that may facilitate the identification of patients with MVP who are at increased risk of ventricular arrhythmias, those with AMVP, and those at risk of SCD [14].

The EHRA committee strongly recommends that ICD implantation be considered in patients with unexplained syncope and high-risk ventricular arrhythmias identified by Holter ECG monitoring, implantable loop recorders (ILRs), or, potentially, exercise testing.

High-risk VAs include sustained VT (>30 s or requiring intervention), VF, and high-risk nsVT, particularly when rapid (>180 beats/min), polymorphic, or associated with unexplained syncope, as well as a malignant PVC phenotype, i.e., malignant ventricular ectopy.

An ILR may help to identify patients with AMVP and high-risk features in the absence of concerning findings on CMR [14].

Patients with MVP who have a documented history of sudden cardiac arrest, VT in the absence of reversible causes, or VF should undergo ICD implantation for secondary prevention [14].

Devices with anti-tachycardia pacing (ATP) capabilities are highly effective in terminating arrhythmias, as evidenced by high success rates (92%) [47]. However, most arrhythmic episodes are PVCs, which can trigger potentially life-threatening VF, whereas monomorphic VT accounts for only a minority of events [32].

The authors of the EHRA consensus on arrhythmic mitral valve prolapse highlighted several clinical scenarios in which ICD implantation may be indicated (Table 3). If pharmacological therapy is ineffective and the arrhythmia remains inadequately controlled by the implanted ICD, patients with recurrent electrical storms should be referred for catheter ablation [14].

### 14.2. Ablation Procedures

Catheter ablation offers an alternative treatment option. Both endocardial and, when necessary, epicardial approaches have demonstrated favorable outcomes in reducing the burden of VT. However, procedural risks, antiarrhythmic drug-related toxicity, and patient preferences must be taken into consideration [14].

VF triggered by complex ventricular ectopy represents a key mechanism of SCD in AMVP. Ablation strategies aim to eliminate PVC trigger sites responsible for the initiation of VF [48].

Ablation may be indicated in patients with PVC-induced cardiomyopathy, VF, or recurrent ICD therapies. Particular caution is required during papillary muscle ablation because of the potential impairment of mitral valve function and the rare risk of left ventricular dysfunction. Scar-related monomorphic re-entrant VT is uncommon and typically originates from the inferolateral or inferobasal segments of the left ventricle [14].

PVCs may arise from multiple ventricular sites, most commonly from the outflow tracts, but also from the mitral annulus and perivalvular regions, particularly in MVP-related arrhythmias. Ablation is recommended in symptomatic patients or those with PVC-induced ventricular dysfunction. Cryoablation may be considered for PVCs originating from the papillary muscles [14].

Mariani et al. conducted a systematic review of the literature using five major databases to assess the efficacy and safety of catheter ablation for ventricular arrhythmias originating from the papillary muscles. The analysis included 356 patients and demonstrated that catheter ablation was an effective treatment for papillary muscle ventricular arrhythmias (PM-VAs), with a relatively low complication rate (1.7%). Acute procedural success after the first ablation procedure was 88.1%, while long-term success following repeat ablation procedures reached 84.9%. The majority of arrhythmias originated from the posteromedial papillary muscle (60.8%) [49].

Nevertheless, the evidence supporting catheter ablation in AMVP is derived predominantly from observational studies and relatively small, heterogeneous cohorts. Differences in the arrhythmic substrate, ablation techniques, procedural endpoints, and follow-up duration limit direct comparisons between studies. Moreover, successful elimination of a dominant PVC focus does not necessarily eliminate the underlying myocardial substrate or the long-term arrhythmic risk.

Ventricular arrhythmias in mitral valve prolapse most often present as focal premature ventricular contractions, but may also include PVC-triggered ventricular tachycardia, ventricular fibrillation, or scar-related re-entrant VT. All of these arrhythmias may be effectively treated with catheter ablation [43,50]. The decision to perform ablation is independent of the MVP phenotype and may be indicated in patients with symptomatic PVCs that are poorly tolerated and do not respond to optimal medical therapy or patients who prefer ablation to long-term drug therapy [14].

Wibawa et al. reported acute procedural success rates ranging from 66% to 94%, with arrhythmia recurrence occurring in 20–30% of patients with MVP [51].

Ablation of MVP-related ventricular arrhythmias should be performed in experienced centers with expertise in both electrophysiology and the management of structural heart disease, including mitral valve pathology [14].

## 15. Surgical Treatment

Extensive long-term data support the efficacy and durability of mitral valve repair for degenerative MVP. Large surgical series have demonstrated excellent long-term outcomes and low reoperation rates following mitral valve repair, including in patients with both classic Barlow disease and fibroelastic deficiency [52,53,54].

In a large prospective cohort of 1234 patients undergoing mitral valve repair for regurgitation due to leaflet prolapse, the cumulative incidence of mitral valve reoperation was only 4.6% at 20 years [53].

These well-established outcomes of surgery for degenerative MVP should, however, be distinguished from the substantially more limited evidence regarding surgical intervention in patients with concomitant MAD, particularly when surgery is considered in relation to ventricular arrhythmic risk. At present, neither MAD nor the presence of arrhythmic risk markers alone constitutes an established indication for mitral valve surgery. Accordingly, decisions regarding surgical intervention in patients with MVP and MAD should continue to follow contemporary recommendations for primary mitral regurgitation, based primarily on mitral regurgitation severity, symptoms, left ventricular function and dimensions, and other established prognostic markers [55].

According to the current ESC/EACTS guidelines, surgery is recommended in symptomatic patients with severe primary mitral regurgitation (MR) who are suitable candidates for surgical intervention. In asymptomatic patients, surgery is indicated when signs of left ventricular dysfunction or remodeling develop, particularly in the presence of an LVEF ≤ 60%, LVESD ≥ 40 mm, or indexed LVESD ≥ 20 mm/m^2^. In asymptomatic patients with preserved left ventricular function, surgery should also be considered in the presence of atrial fibrillation secondary to MR or pulmonary hypertension with a resting systolic pulmonary artery pressure (SPAP) > 50 mmHg. Furthermore, in selected patients at low surgical risk and with a high likelihood of durable mitral valve repair, significant left atrial enlargement (LAVI ≥ 60 mL/m^2^ or left atrial diameter ≥ 55 mm) may support consideration of earlier surgical intervention [55].

Beyond these conventional indications, increasing attention has focused on whether mitral valve surgery might also modify the arrhythmic phenotype of MVP. In selected patients, particularly those with a history of complex VA, concomitant surgical cryoablation during mitral valve surgery has been proposed as a potentially effective therapeutic strategy [14].

The effect of mitral valve surgery on arrhythmic burden following valve repair remains unclear. A study of 599 patients who underwent mitral valve surgery suggested that the risk of ventricular arrhythmias remained high despite surgery. Of these patients, 485 underwent mitral valve repair and 114 underwent mitral valve replacement. Of the 599 patients, 96 had been diagnosed with MAD before surgery. Each 1 mm increase in mitral annular disjunction length was associated with a 35% increase in the risk of postoperative VA.

Patients with MAD were, on average, approximately 8 years younger, with a higher proportion of women (31% vs. 17%) and a higher prevalence of Barlow’s disease (70% vs. 27%). Despite surgical correction of MAD, its presence in patients with MVP and mitral regurgitation was associated with a threefold higher long-term risk of postoperative ventricular arrhythmias [20].

Cameron et al. reviewed 16 studies (8 case reports and 8 cohort studies), including a total of 1233 patients, of whom 657 with MVP underwent mitral valve surgery. Seven cohort studies reported the incidence of VA before and after surgery, six of which demonstrated a significant reduction. The remaining cohort study reported a reduction in the risk of SCD following surgical treatment. A reduction in the risk of VA following mitral valve surgery was also reported in all case reports [41].

According to the data reported by Markeviciute et al. regarding surgical mitral valve repair in arrhythmic MVP, the incidence of ventricular arrhythmias decreased in more than half of the patients (55%). However, de novo ventricular arrhythmias occurred postoperatively in 19% of patients who had not previously experienced them. It remains unclear which factors predispose patients to the development of ventricular arrhythmias after surgical intervention. Surgery may therefore both reduce the arrhythmic burden and unmask latent arrhythmogenic substrates [40].

The potential antiarrhythmic effect of mitral valve surgery in arrhythmic mitral valve prolapse (AMVP) remains uncertain. Successful mitral valve repair may reduce abnormal mechanical traction on the papillary muscles and adjacent inferobasal myocardium. Correction of mitral regurgitation, restoration of annular geometry, and reduction of MAD-related systolic motion may therefore decrease mechanical triggers for ventricular ectopy. However, whether surgery modifies the established arrhythmogenic substrate remains unclear. Replacement fibrosis involving the papillary muscles and peri-annular or inferobasal myocardium may persist after successful valve repair and continue to provide a substrate for ventricular arrhythmias [56].

Available clinical data suggest that mitral valve surgery may reduce ventricular arrhythmic burden in some patients. In a prospective study of patients with Barlow’s disease undergoing mitral valve repair, 55% of patients with significant preoperative arrhythmic burden no longer fulfilled arrhythmic criteria at follow-up. However, 45% remained arrhythmogenic, while 18.6% of patients without significant preoperative ventricular arrhythmias developed a significant arrhythmic burden after surgery. These findings highlight the heterogeneous electrophysiological response to mitral valve repair and indicate that correction of valve mechanics does not uniformly eliminate arrhythmic susceptibility [20].

However, the evidence remains limited by its predominantly observational nature, small sample sizes, heterogeneous arrhythmic endpoints, and variable monitoring and surgical strategies. Importantly, residual ventricular arrhythmias and SCD have been reported despite successful surgery, indicating that mitral valve repair should not be considered intrinsically curative for the arrhythmic phenotype [41].

An important unresolved question is whether earlier mitral valve repair could prevent progression from a mechanically mediated arrhythmic trigger to an established fibrotic substrate. Currently, there is insufficient evidence to recommend surgery solely to prevent ventricular arrhythmias or SCD in patients with AMVP without conventional indications for intervention. Although expert consensus suggests that surgery may reduce malignant ventricular arrhythmias in patients with MVP and severe mitral regurgitation, this is based predominantly on observational evidence and expert opinion. Whether patients with a pronounced arrhythmic phenotype might benefit from earlier intervention remains unknown and warrants prospective investigation [20].

Taken together, current evidence suggests that mitral valve surgery may reduce arrhythmic burden in selected patients; however, the predominantly observational data do not support mitral valve repair as an arrhythmia-directed intervention in the absence of conventional surgical indications. Whether earlier surgery modifies the arrhythmogenic substrate and reduces long-term SCD risk remains unresolved.

## 16. Conclusions

The pathophysiology of potentially lethal ventricular arrhythmias associated with MVP remains incompletely understood and requires further investigation. The occurrence of SCD in patients with arrhythmic MVP is associated with specific high-risk features, including bileaflet MVP, MAD, the Pickelhaube sign, T-wave inversion on ECG, and rapid non-sustained VT. From a clinical perspective, identification of these features should prompt comprehensive multimodal risk assessment integrating clinical presentation, ECG and extended rhythm monitoring, echocardiography, and CMR, particularly for the detection and characterization of myocardial fibrosis. Patients with MVP, with or without MAD, who are at increased risk of ventricular arrhythmias should undergo regular clinical and rhythm monitoring. Importantly, no single clinical or imaging marker is currently sufficient to reliably predict malignant ventricular arrhythmias or SCD, highlighting the importance of integrated risk stratification and individualized clinical decision-making.

The prognosis and optimal management of arrhythmic MVP remain uncertain and warrant further well-designed multicenter studies. Future research should focus on prospective validation of multiparametric risk-stratification models; the prognostic significance and quantitative assessment of myocardial and papillary muscle fibrosis, including LGE burden and parametric mapping; and the identification of clinically meaningful thresholds for imaging and arrhythmic markers. Further studies are also needed to clarify the contribution of genetic susceptibility and identify high-risk patients who may benefit from preventive interventions, including catheter ablation, mitral valve surgery, or primary-prevention ICD implantation.

## Figures and Tables

**Figure 1:**
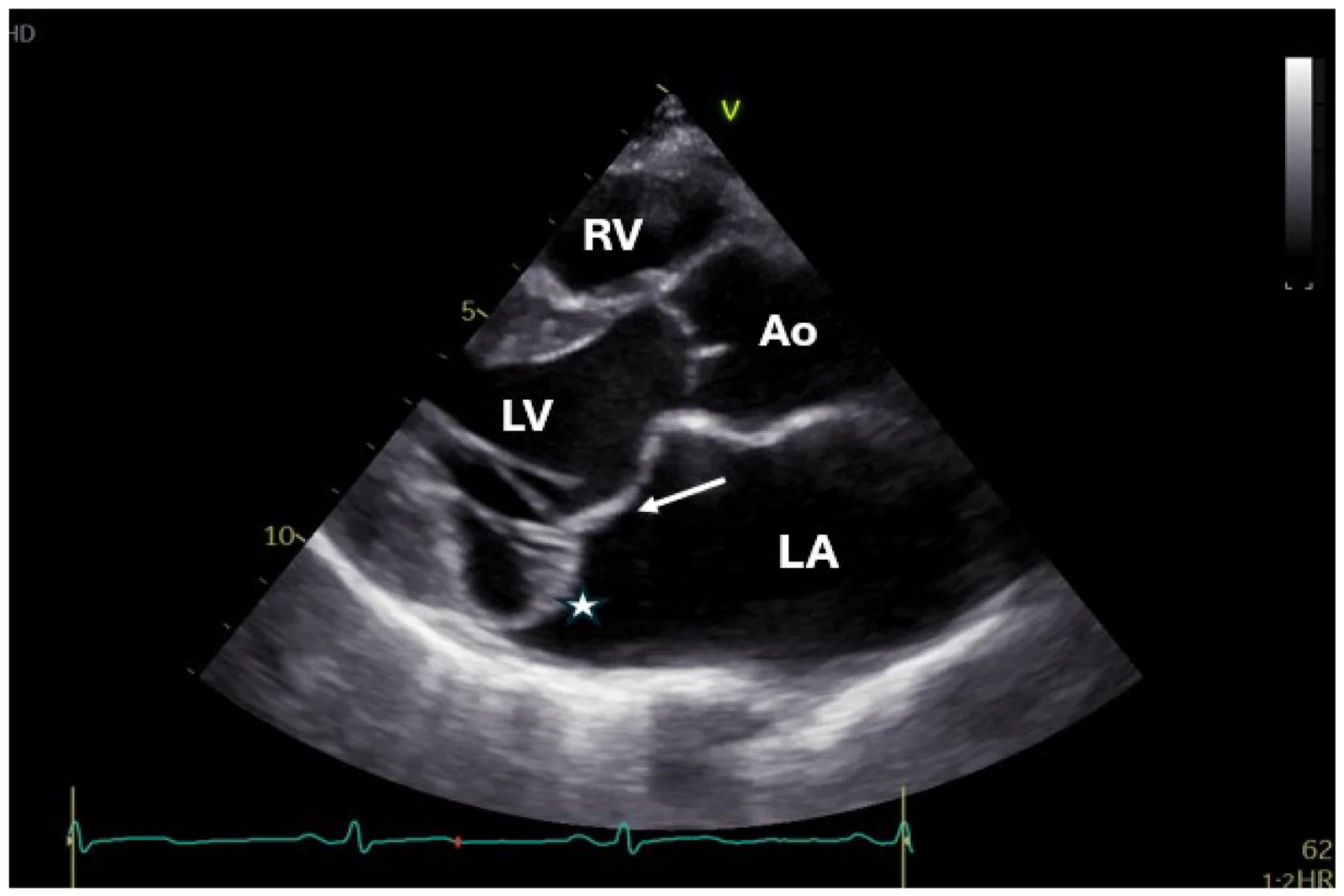
TTE parasternal long-axis view—bileaflet mitral valve prolapse (white arrow—anterior leaflet, white star—posterior leaflet). Ao—aorta, LA—left atrium, LV—left ventricle, RV—right ventricle.

**Figure 2:**
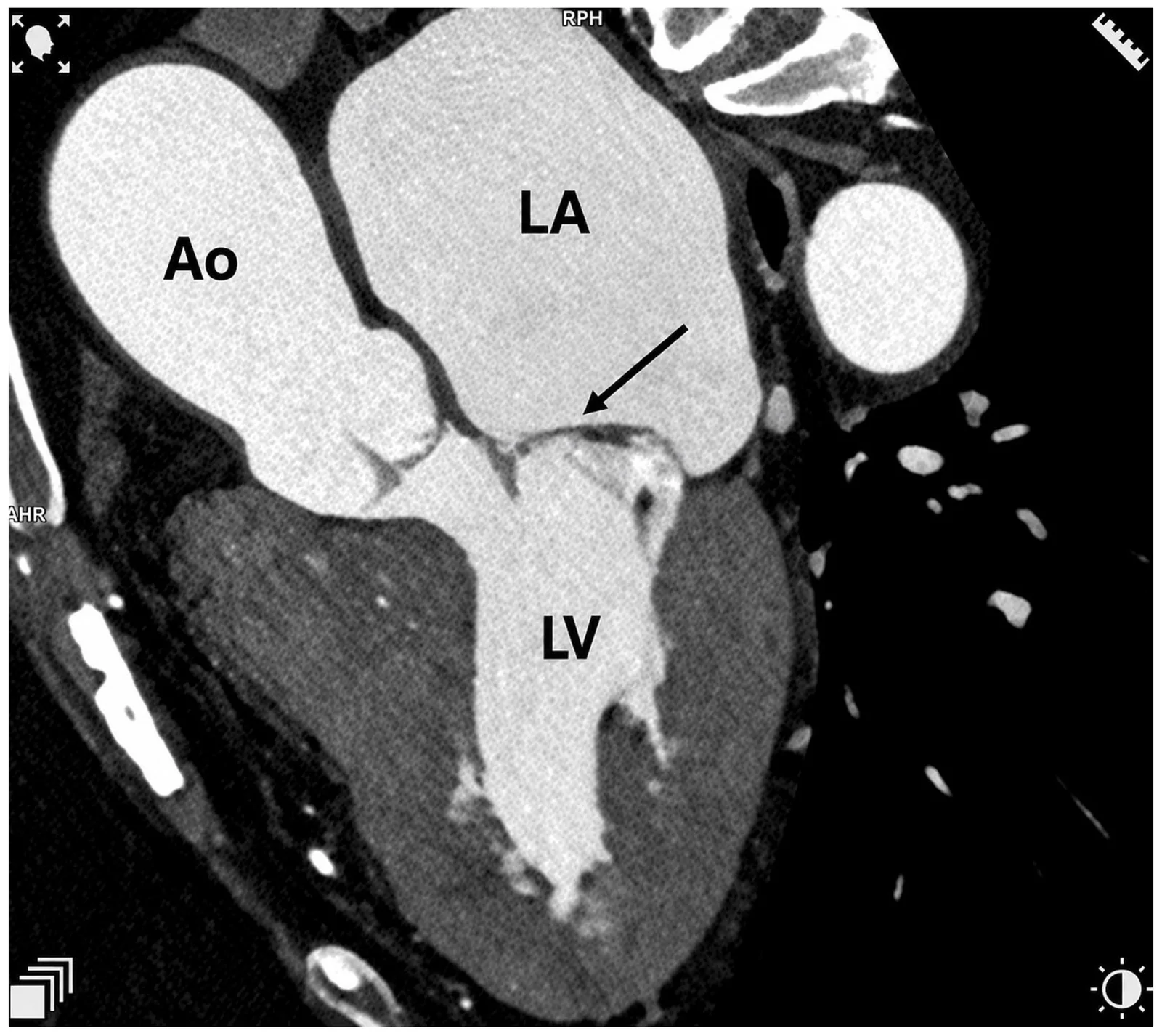
Heart CT—three-chamber view; posterior leaflet prolapse (black arrow). Ao—aorta, LA—left atrium, LV—left ventricle.

**Figure 3:**
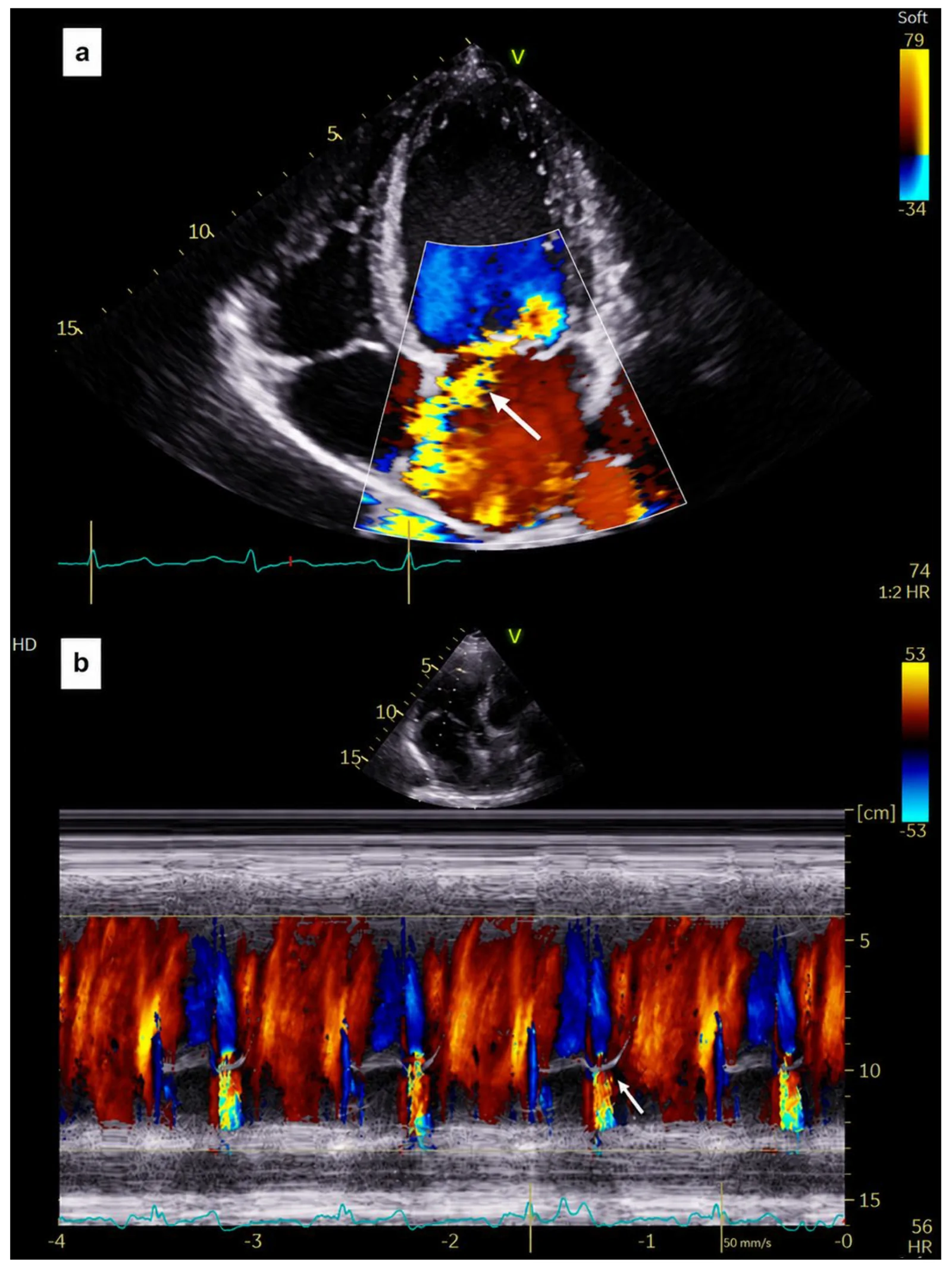
Transthoracic echocardiography, (**a**) apical four-chamber view, color Doppler, mitral regurgitation with eccentric jet (white arrow). (**b**) Color M-mode, late systolic regurgitation (white arrow).

**Figure 4:**
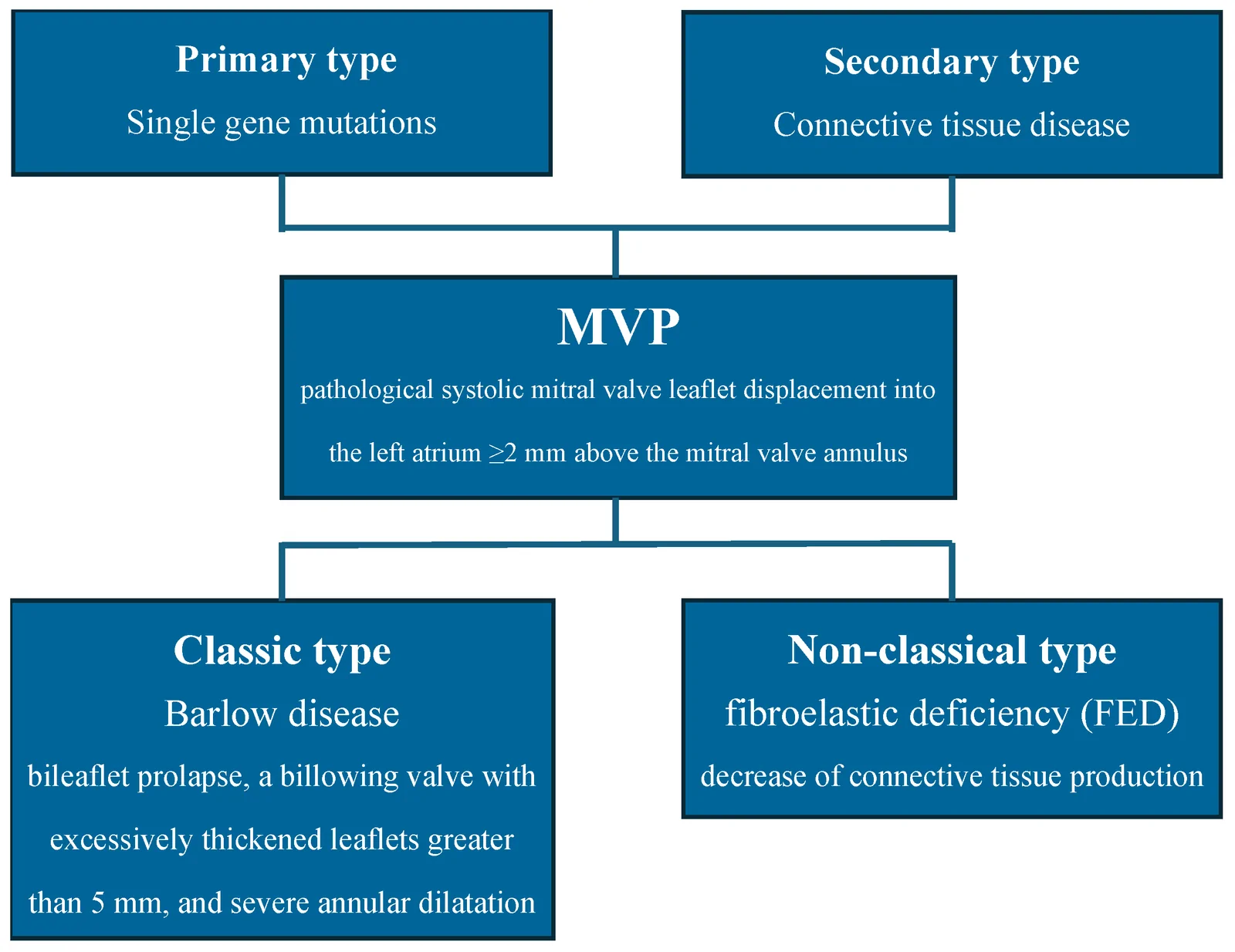
Classification of MVP according to the etiology and pathophysiology of the syndrome [10].

**Figure 5:**
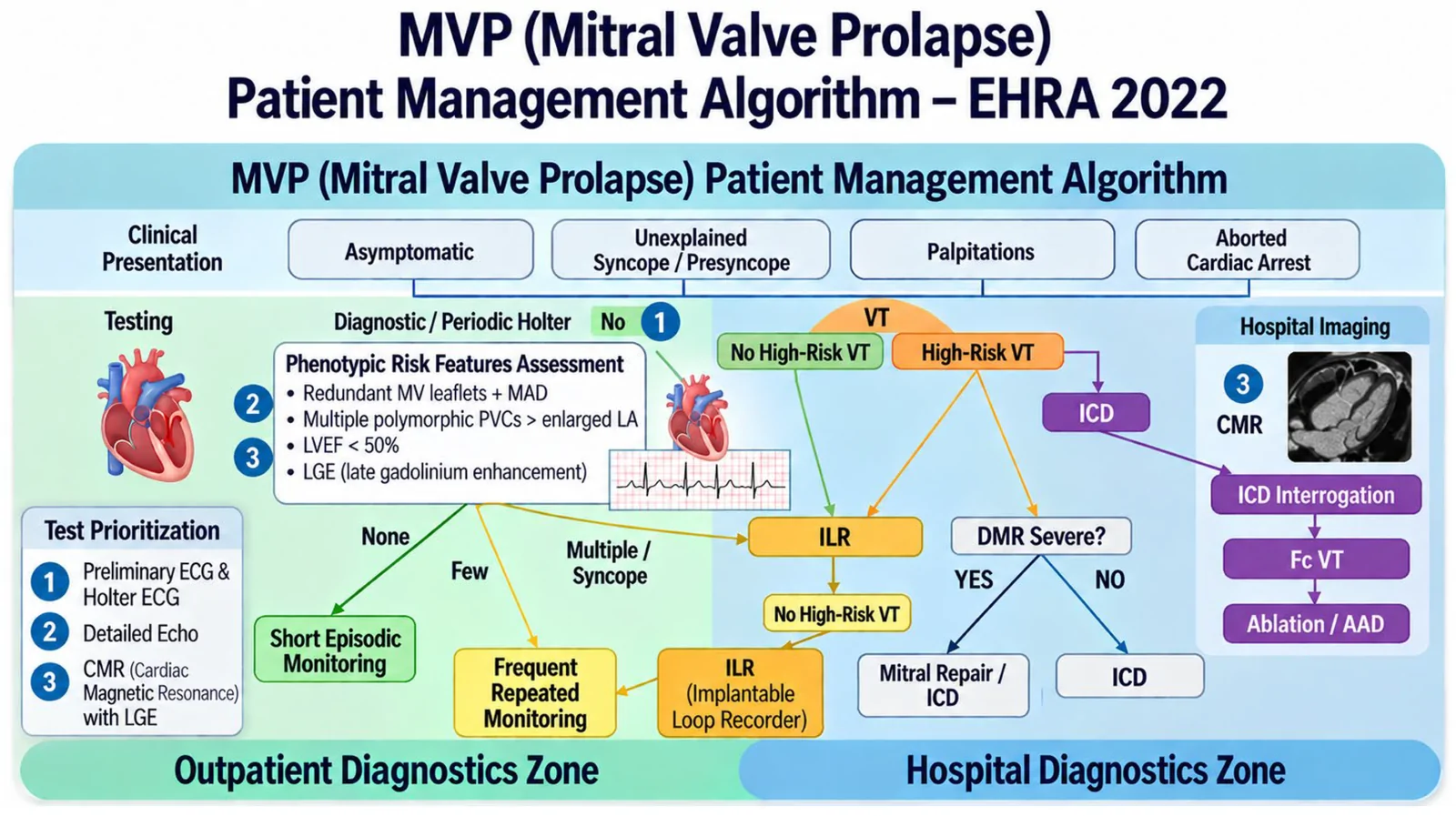
EHRA expert consensus statement on arrhythmic mitral valve prolapse and mitral annular disjunction complex, 2022 [14]. The figure shows the treatment regimen for MVP patients, divided into outpatient and hospital diagnostics. VT—ventricular tachycardia, PVCs—premature ventricular contractions, MV—mitral valve, MAD—mitral annular disjunction, LA—left atrium, LVEF—left ventricular ejection fraction, LGE—late gadolinium enhancement, ILR—implantable loop recorder, DMR—degenerative mitral regurgitation, ICD—implantable cardioverter-defibrillator, AAD—antiarrhythmic drug therapy.

**Figure 6:**
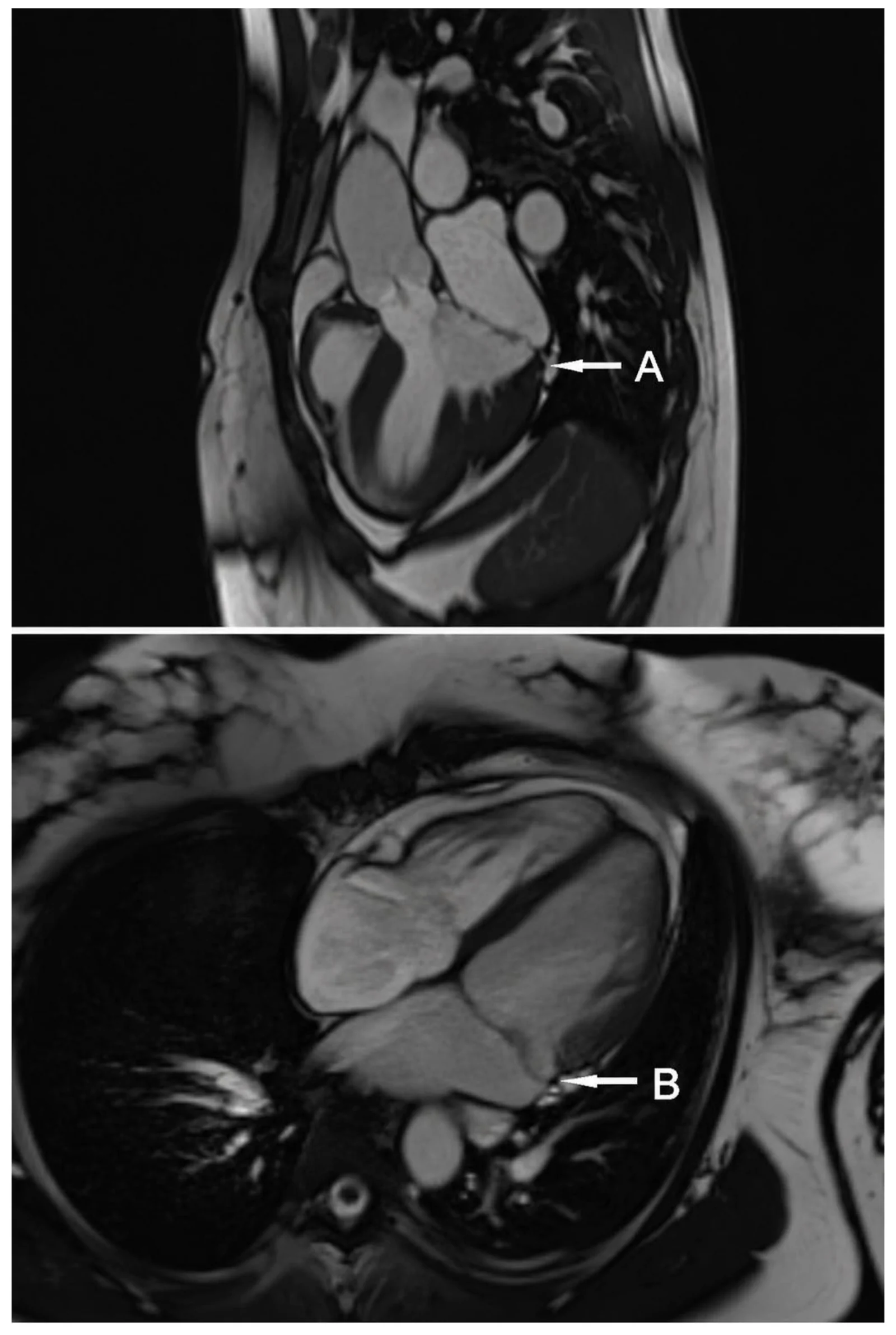
Cine CMR, mitral annulus disjunction (MAD) of 4 mm in the three-chamber (**A**) and four-chamber (**B**) views.

**Figure 7:**
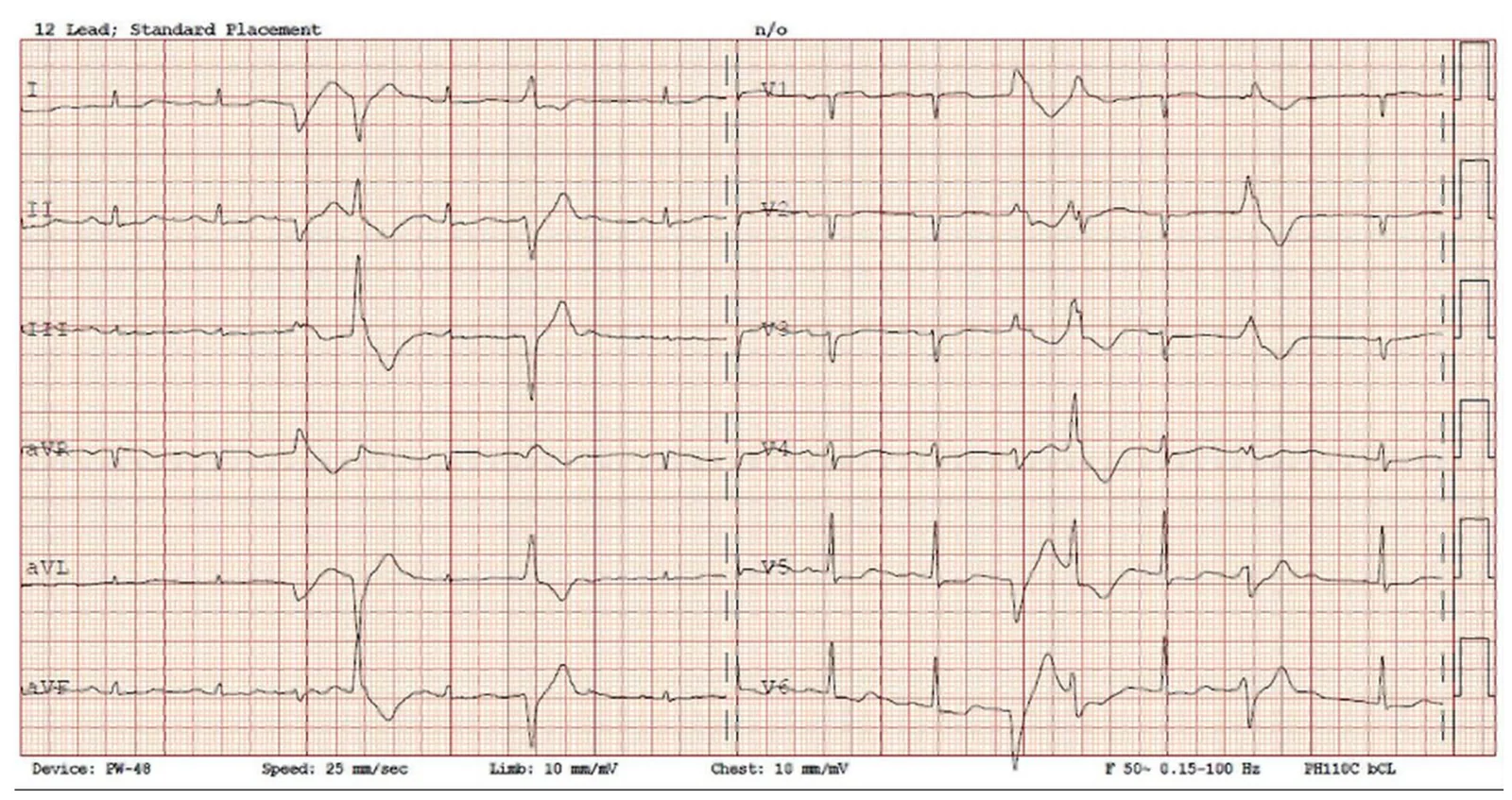
Arrhythmia in a 33-year-old female with MAD and a history of aborted sudden cardiac arrest. Sinus rhythm with polymorphic premature ventricular couplets and complexes.

**Figure 8:**
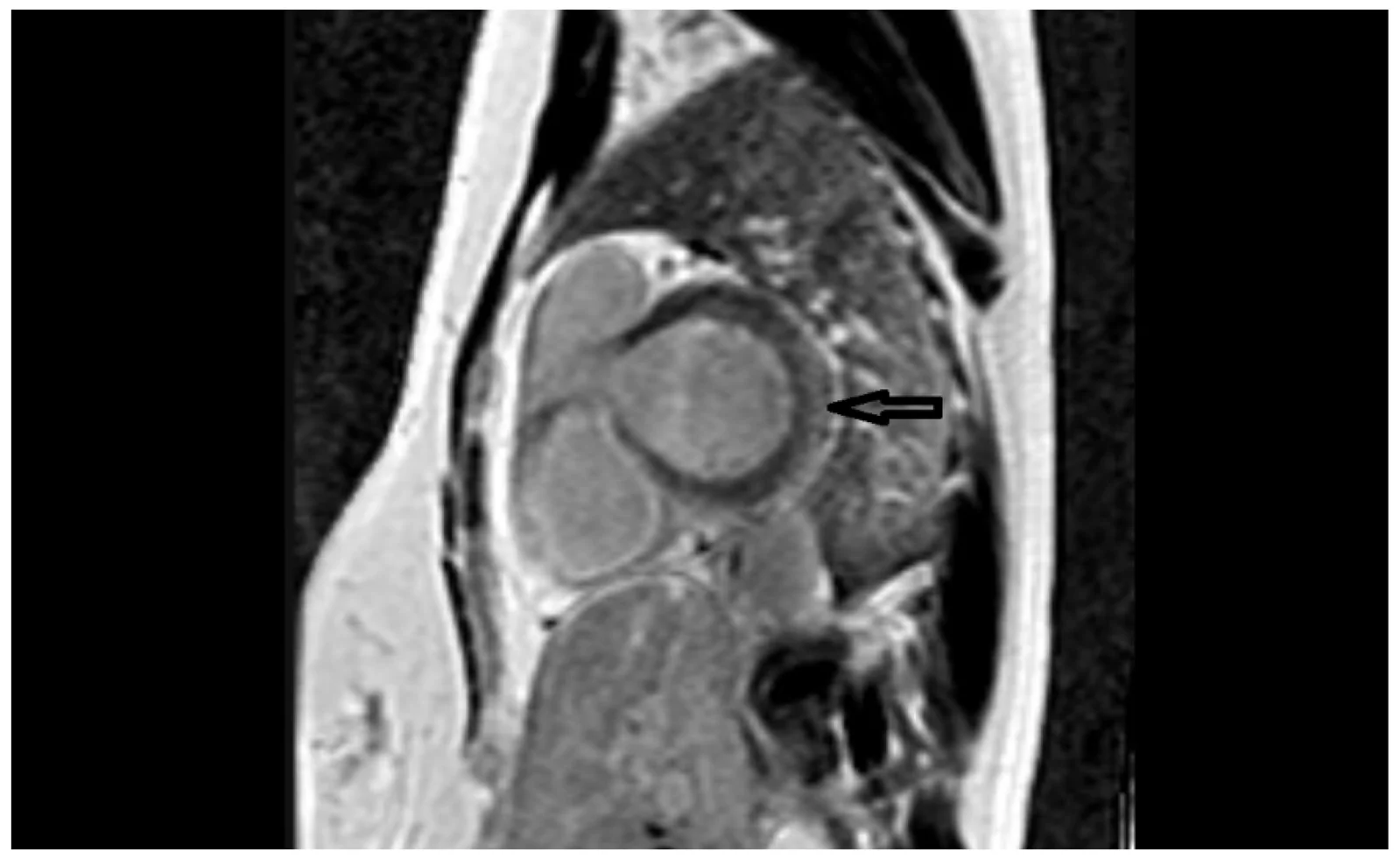
CMR, short-axis view. Late gadolinium enhancement (the arrow) indicates myocardial fibrosis.

**Table 1:** Clinical, electrocardiographic, echocardiographic, cardiac magnetic resonance, and genetic markers associated with arrhythmic risk in patients with MVP.

Category	Risk Marker	Association with Arrhythmic Risk
Clinical markers	Unexplained syncope or presyncope	Associated with an increased likelihood of clinically relevant ventricular arrhythmias; should prompt intensified rhythm monitoring and further risk assessment
	History of sustained VT, VF, or aborted SCA	Defines a very high-risk phenotype and constitutes an indication for secondary-prevention strategies, including ICD implantation
	Family history of sudden cardiac death	May indicate increased arrhythmic susceptibility, particularly in selected AMVP phenotypes
	Younger age	Reported in association with arrhythmic MAD/AMVP phenotypes and ventricular arrhythmias
ECG markers	T-wave inversion/repolarization abnormalities	Recognized as a phenotypic marker of arrhythmic MVP and may warrant extended rhythm monitoring
Holter/rhythm-monitoring markers	Frequent PVCs/PVC burden > 5%	Frequent ventricular ectopy is characteristic of AMVP and may indicate an active arrhythmogenic substrate
	Complex or polymorphic ventricular ectopy	May indicate a more arrhythmogenic phenotype and should prompt further risk assessment
	Fast or polymorphic nsVT	Represents a high-risk arrhythmic feature, particularly when rapid or associated with unexplained syncope
	Short-coupled PVCs	May act as triggers for malignant ventricular arrhythmias, including VF
Echocardiographic markers	Mitral annular disjunction (MAD)	Frequently associated with ventricular arrhythmias and arrhythmic MVP; greater MAD extent may indicate higher risk
	MAD distance > 8.5 mm	Reported as a marker associated with an increased risk of ventricular arrhythmias
	Bileaflet MVP/redundant leaflet tissue	Commonly observed in arrhythmic MVP and in patients with unexplained cardiac arrest
	Pickelhaube sign	More frequently observed in MVP patients who survived sudden cardiac arrest
	Systolic atrial-directed notch (SADN) > 2 mm	Reported more frequently in patients with MVP after sudden cardiac arrest
	Reduced LVEF	Associated with adverse outcomes and may identify a higher-risk structural phenotype
	Abnormal longitudinal strain/mechanical dispersion	May reflect abnormal myocardial mechanics associated with fibrosis and ventricular arrhythmias
CMR markers	Presence of LGE	Marker of myocardial replacement fibrosis and a potential arrhythmogenic substrate
	Papillary muscle LGE/fibrosis	Particularly relevant in AMVP and associated with ventricular arrhythmias
	Basal inferolateral/inferior LV fibrosis	Typical distribution of fibrosis in AMVP and associated with arrhythmogenic remodeling
	Greater LGE burden/more extensive fibrosis	May provide incremental information beyond binary LGE assessment, although validated prognostic thresholds are lacking
	Increased native T1/extracellular volume (ECV)	May identify diffuse interstitial fibrosis not detectable by conventional LGE and could improve phenotypic characterization
	MAD detected by CMR	Provides structural characterization of the mitral annulus and may complement echocardiographic risk assessment
Genetic markers	Familial MVP/pathogenic variants in FLNA, DCHS1, DZIP1, and PLD1	Associated with inherited MVP susceptibility, although their value for individual arrhythmic risk prediction remains uncertain
	Syndromic connective-tissue disease	MVP may occur in inherited connective-tissue disorders, but their independent contribution to arrhythmic risk remains insufficiently defined

**Table 3:** Potential indications for ICD implantation in patients with arrhythmic MVP.

Recommendation Category	Clinical/Imaging Criteria
**Implantation may be justified**	Unexplained syncope and sustained ventricular tachycardia with possible origin from the mitral valve apparatus (MVA)
	Unexplained syncope and non-sustained ventricular tachycardia (nsVT) probably arising from MVA
**Implantation can be discussed**	Patients with one high-risk factor (unexplained syncope and/or nsVT) and at least two morphological changes:
	T-wave inversions in inferior ECG leads
	Multiple polymorphic ventricular extra beats
	Mitral annular disjunction
	Enlarged left atrium
	Left ventricular ejection fraction ≤ 50%
	Late gadolinium enhancement (LGE) on CMR

## Data Availability

No original data were created or analyzed in this study. Data sharing is not applicable to this article.
